# Microscopic examination of pituitary glands in cases of fatal accidental hypothermia

**DOI:** 10.1080/20961790.2017.1330804

**Published:** 2017-06-07

**Authors:** Elke Doberentz, Burkhard Madea

**Affiliations:** Institute of Forensic Medicine, University of Bonn, Bonn, Germany

**Keywords:** Forensic pathology, adenohypophysis, haemorrhage, hypothermia, hyperaemia, pituitary gland, vacuolization, post-mortem examination

## Abstract

In cases of death caused by hypothermia, histological analysis can be used to determine the cause of death. Certain histological alterations of the pituitary glands in hypothermia have been reported in the literature, including haemorrhage, hyperaemia and cellular vacuolization of cells in the anterior lobe. In the present study, the validity of these morphological alterations as markers for fatal accidental hypothermia was investigated in autopsy material. A total of 34 pituitary glands in cases of verified fatal accidental hypothermia were examined histologically (haematoxylin and eosin, ferric, azan) and immunohistochemically (LCA, ACTH, C5b-9). The findings were compared with 61 cases in a control group. Hyperaemia was found in 50.0% of the study group cases and 59.0% of the control group cases. Cellular vacuolization was observed in one case (2.9%) in the study group and one case (1.6%) in the control group. Acute or recent haemorrhage in the glandular tissue was never detected. In our study, the histopathological characteristics described in the literature as pathognomonic for hypothermia could not be confirmed. Furthermore, histological differences in the pituitary glands between fatal hypothermia cases and control group cases were not observed.

## Introduction

A core body temperature below 35 °C is defined as hypothermia [1,2]. In this condition, heat loss from the body exceeds heat production by the body. Temperature receptors send signals to the hypothalamus to activate physiological mechanisms that maintain the core body temperature at the physiological level of 36–37 °C [3,4]. The hypothalamus also regulates hormone-producing cells in the pituitary gland. The hormones produced in the anterior lobe of this endocrine gland are adrenocorticotropic hormone (ACTH), follicle-stimulating hormone, luteinising hormone, thyroid-stimulating hormone (TSH), melanocyte-stimulating hormone, prolactin and growth hormone. Together, these hormones regulate hormone release by peripheral endocrine glands, such as the adrenal gland and thyroid gland, and have a direct influence on body metabolism. In a hypothermic state, up-regulation of body metabolism is the first step in elevating the core body temperature.

It has been established that hypothermia, a condition of systemic stress, activates the adenohypophysis. Recently, the adenohypophysis has become a focus of medico-legal research to establish whether typical quantifiable changes occur in response to pre-mortal thermal influences [5–13]. This is of importance because the usually sparse manifestations of macromorphological and micromorphological findings in fatal hypothermia cases make it difficult to diagnose the cause of death [14,15].

The following histological findings of the pituitary gland anterior lobe have been described in cases of fatal hypothermia: hyperaemia, haemorrhage and cytoplasmic vacuolization of cells (Table 1) as well as inflammatory reactions in the cerebral tissue [16].

**Table 1.** Reported findings for the adenohypophysis in cases of fatal hypothermia versus control cases in the literature.

	Hyperaemia		Haemorrhage		Vacuolization	
Authors	Study	Control	Study	Control	Study	Control
Verdiccio M et al. (2006) [9]	NR	NR	81.8%	0%	NR	NR
Ishikawa T et al. (2004) [8]	NR	NR	NR	NR	40%	1%
Hirano N et al. (1994) [7]	NR	NR	NR	NR	P	NR
Baillif RN (1944) [5]	P	NR	NR	NR	P	NR
Büchner F (1943) [6]	NF	NR	NF	NR	NF	NR
Present study	50.0%	59.0%	0%	0%	2.9%	1.6%

Note: In the public literature reported findings in the adenohypophysis in cases of fatal hypothermia vs. a control group (NR: not reported, P: positive results and NF: not found in the examination).

The aim of the present study was to investigate fatal hypothermia cases in our archived autopsy material with regard to the above-mentioned morphological changes. The study is a follow-up study to our recently published paper [10] investigating micromorphological alterations in the pituitary gland anterior lobe in comparison with a control group without pre-mortal thermal influences or head trauma in a larger sample size collected prospectively.

## Materials and methods

We re-analysed the histological material from our previously published study [10] together with 17 prospectively sampled cases, giving a total of 34 fatal hypothermia cases. The study group comprised 19 females and 15 males aged between 9 and 97 years (mean: 66.5 years). The deceased females were aged between 31 and 97 years (mean: 70.8 years) and the deceased males were aged between 9 and 87 years (mean: 61.3 years). The victims were found indoors in 24 cases, and outdoors in 10 cases.

In all cases, typical findings of hypothermia were observed (Table 2) and competitive causes of death could be excluded. The details for the study group (age, sex, place of discovery, macroscopic and microscopic findings) are shown in Table 3. The actual time of exposure was not known, and varied from 1.5 to about 12 hours.

**Table 2.** Findings for hypothermia in the study group (*N* = 34).

Findings of hypothermia	Number of cases (%)
Wischnewsky spots	33 (97.06)
Cold erythema (purple discoloration over knees and large joints)	14 (41.17)
Paradoxical undressing	5 (14.7)
Hiding	1 (2.94)
Haemorrhage into the M. psoas major	1 (2.94)

**Table 3.** Details for the 34 cases of fatal hypothermia in the study group with macroscopic and microscopic findings.

No.	Age	Sex	Place of discovery	Wischnewski spots	Frost erythema	Paradoxical undressing	Hiding	Psoas hemorrhage	Hyperamia	Hamorrhage	Vacuolization	LCA	C5b-9	ACTH
1	88	W	Indoor	X										X
2	87	W	Outdoor	X										X
3	80	M	Outdoor	X	X									X
4	91	W	Indoor	X	X									X
5	59	W	Indoor	X					X					X
6	78	W	Indoor	X		X	X		X					X
7	60	M	Indoor	X		X								X
8	60	M	Indoor	X	X				X					X
9	43	M	Indoor	X								X		X
10	92	W	Indoor	X	X						X			X
11	44	M	Outdoor	X		X			X					X
12	44	W	Outdoor	X	X				X					X
13	9	M	Indoor	X					X			X		X
14	63	M	Indoor	X	X	X			X					X
15	88	W	Outdoor		X	X			X					X
16	31	W	Outdoor	X		X			X					X
17	55	W	Indoor	X		X			X					X
18	58	W	Indoor	X										X
19	57	M	Outdoor	X					X					X
20	62	W	Indoor	X										X
21	78	W	Indoor	X										X
22	80	W	Outdoor	X					X					X
23	53	W	Outdoor	X										X
24	76	M	Indoor	X										X
25	61	M	Indoor	X										X
26	66	M	Outdoor	X	X									X
27	71	M	Indoor	X	X				X					X
28	67	M	Indoor	X	X				X					X
29	76	M	Outdoor	X	X	X								X
30	50	W	Outdoor	X					X					X
31	71	W	Indoor	X	X			X	X					X
32	97	W	Indoor	X	X									X
33	87	M	Indoor	X					X					X
34	83	W	Indoor	X	X									X

Note: Study group 34 cases of death due to hypothermia with macro- and microscopical findings.

LCA: leucocyte common antigen; C5b-9: C5b-9 complement membrane complex; ACTH: adrenocorticotropic hormone; X: positive.

The control group consisted of 61 pituitary glands in cases with natural and non-natural causes of death without known ante-mortem thermal stress or head trauma. This group included 26 females and 35 males, with a mean age of 58.3 years (range: 7–100 years). The females had a mean age of 69.8 years (range: 7–88 years) and the males had a mean age of 48.6 years (22–100 years). The causes of death listed in alphabetical order are presented in Table 4.

**Table 4.** Causes of death in the 61 cases in the control group listed in alphabetical order.

Cases of death	Number of cases (%)
Bolus death	1 (1.6)
Burking	1 (1.6)
Cachexia	1 (1.6)
Cardiac failure	3 (4.9)
Drowning	1 (1.6)
Drug intoxication	11 (18.0)
Flue gas intoxication	2 (3.3)
Haemorrhage	8 (13.1)
Hypertensive crisis	1 (1.6)
Hypoxic brain damage	2 (3.3)
Mors in tabula	1 (1.6)
Myocardial infarction	14 (23.0)
Pancreatitis	1 (1.6)
Pericardial tamponade due to ruptured aortic aneurysm	2 (3.3)
Polytrauma (without head trauma)	1 (1.6)
Pneumonia	1 (1.6)
Pulmonary embolism	4 (6.6)
Pulmonary oedema due to congestive heart failure	1 (1.6)
Sepsis	1 (1.6)
Strangulation	3 (4.9)
Stroke (cerebral infarction)	1 (1.6)

Note: Causes of death in the control group listed in alphabetical order (*N* = 61).

All pituitary glands were collected during autopsy. The formalin-fixed pituitary glands were cut in the sagittal plane to obtain a representative cross-section through the anterior and posterior lobes. The tissue was then embedded in paraffin wax. The samples were subjected to routine staining procedures, such as haematoxylin and eosin, azan and ferric staining, to detect hyperaemia, haemorrhage, signs of previous haemorrhage and cellular vacuoles as a sign of hypoxidosis. In addition, immunohistochemical staining for leucocyte common antigen (LCA) was performed to detect accumulation of leucocytes as a sign of inflammation after long-lasting hypoxidosis.

The pituitary glands in the entire control group and a subset of 17 fatal hypothermia cases (from our previous study group [10]) were subjected to immunohistochemical staining for ACTH and C5b-9 (C5b-9 complement membrane complex), a marker for necrosis. The following antibodies and dilutions were used: anti-CD45 antibody [MEM-28] (ab8216; Abcam; dilution 1:200); anti-C5b-9 antibody (ab55811; Abcam; dilution 1:100); anti-ACTH antibody (ab74976; Abcam; dilution 1:200); and secondary antibody (EnVision™+ Dual Link System-HRP; Dako). All pituitary gland sections were analysed by light microscopy. The examinations were carried out by board-qualified forensic pathologists.

## Results

**Figure 1.**
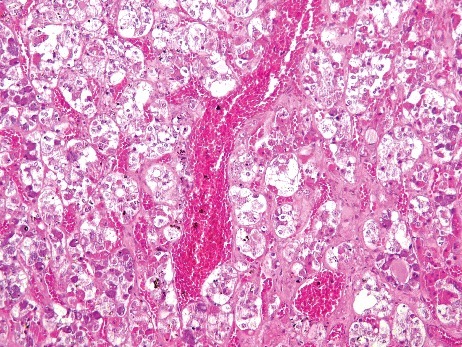
Hyperaemic tissue of the adenohypophysis in a case of fatal hypothermia (H&E × 200).

Signs of hyperaemia and acute or recent haemorrhage were analysed based on haematoxylin and eosin, azan and ferric staining. Hyperaemia was observed in 17 of 34 pituitary glands (50.0%) in the study group (Figure 1). Similarly, 36 of 61 pituitary glands (59.0%) in the control group presented signs of hyperaemia within the anterior lobe. Haemorrhage or ferric staining was not observed in either the study group or the control group.

**Figure 2.**
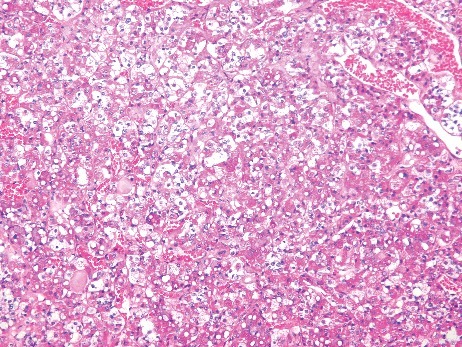
Vacuolization in the adenohypophysis in a case of fatal hypothermia (H&E × 200).

**Figure 3.**
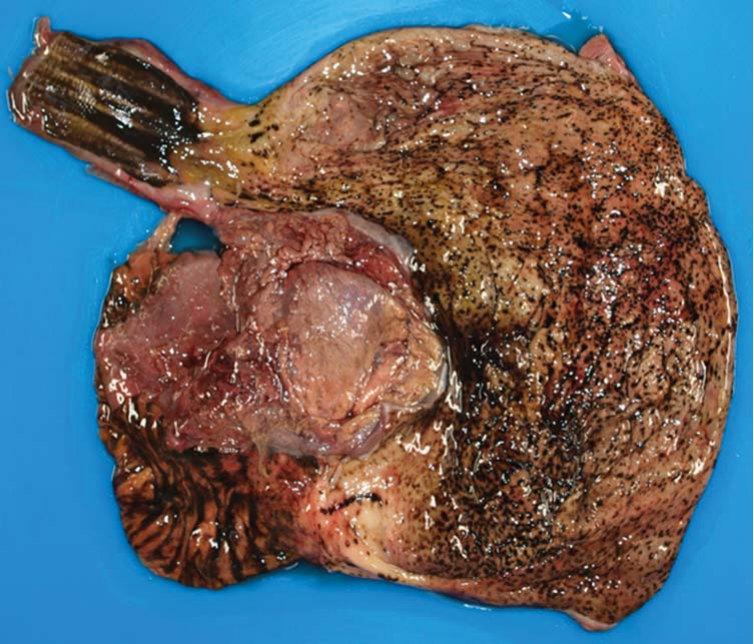
Wischnewsky spots in the gastric mucosa in a case of fatal hypothermia.

**Figure 4.**
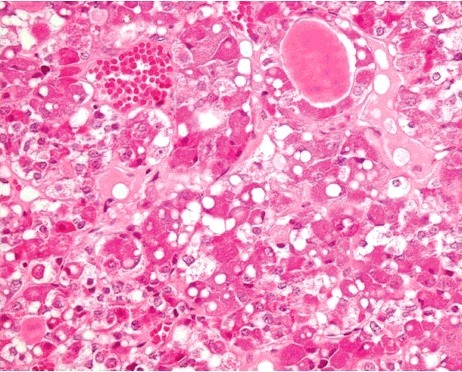
Vacuolization of the anterior lobe of the pituitary gland in a control case (H&E × 400).

Vacuolization proved difficult to evaluate because of autolytic alterations to the tissue with the beginning of disappearance of cell structures. Vacuolization was observed in only one case (2.9%) in the study group (Figure 2). This case was a 92-year-old woman who died in her apartment. As macromorphological signs, Wischnewsky spots (Figure 3) and cold erythema were present. Furthermore, only 1 case (1.6%) in the control group had small cytoplasmic vacuoles in the anterior pituitary cells (Figure 4).

**Figure 5.**
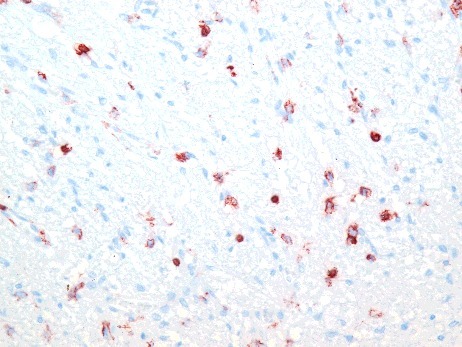
LCA-positive cells in the anterior lobe of the pituitary gland in a case of fatal hypothermia (×400).

**Figure 6.**
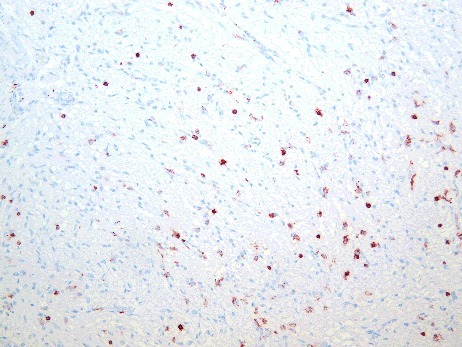
LCA-positive reaction in the adenohypophysis in a control case (×200).

Immunohistochemical analysis of LCA revealed positively-stained leucocytes in the anterior lobe in two cases (5.9%) in the study group (Figure 5). In contrast, 25 cases (41.0%) in the control group had LCA-positive leucocytes within the tissue (Figure 6).

In our previous study [10], immunohistochemical staining for C5b-9 as a necrosis marker did not reveal positive results for any of the study cases or control cases. Thus, C5b-9 staining was not continued. ACTH immunostaining was positive in all cases in both groups with similar expression patterns; however, autolytic changes to the tissue prevented any possibility of quantification.

**Figure 7.**
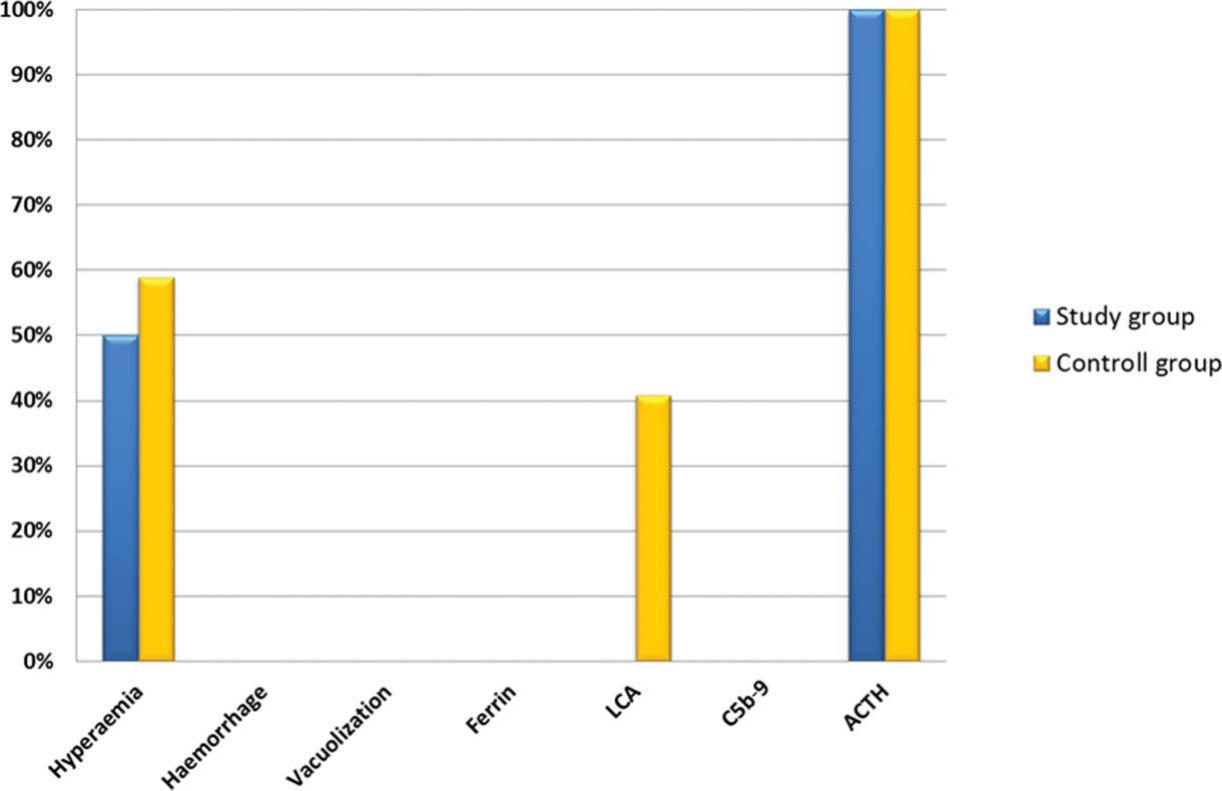
Summary of all results in the study group and control group as percentages.

A summary of all results for the study group and control group is provided in Figure 7. Overall, this follow-up study confirmed the results of our previous study in a larger sample size.

## Discussion

In cold environmental conditions, homeothermic organisms aim to keep their core body temperature at a physiological level between 36 and 37 °C. A drop in the core body temperature below 32 °C, and especially down to 28 °C, increases the risk of myocardial dysfunction with hypotonia, atrial fibrillation with reduced cardiac output and asystole. Finally, a core body temperature below 25 °C is lethal [3,17–21].

A drop in the core body temperature leads to cardiovascular dysfunction and hypoxidosis, which induces cellular stress and alterations in several organs that can be analysed histologically and immunohistochemically:

• Colloid depletion and increase in thyroid gland epithelium [1,22].
• Vacuolization and fatty degeneration in myocytes and hepatocytes [22–24].
• Depletion of glycogen in myocytes, skeletal muscle cells and hepatocytes [20,22].
• Pancreatic haemorrhage, acute pancreatitis and vacuolization in pancreatic cells [25].
• Vacuolization and fatty degeneration in renal tubular epithelium [26,27].
• Increased myoglobin level in renal tubular epithelium and increased ubiquitin level in distal renal tubules and collecting ducts [28].
• Increased heat shock protein expression in the cerebral cortex [29].
• Decreased ACTH and TSH expression in the adenohypophysis [11,30].
• Increased expression of heat shock proteins in renal tissue [31].

While the appearance of histological findings alone is not specific for hypothermia, accumulation of several of these markers can help to verify the diagnosis when hypothermia is suspected.

In particular, vacuolization and fatty degeneration in renal tubular epithelium as a diagnostic marker in fatal hypothermia cases has been confirmed by several authors [26,27]. It was reported that alterations to the tissue in the anterior lobe of the pituitary gland are involved in thermoregulation. Hyperaemia of the glandular tissue was reported by Bailif [5]. In the present study, hyperaemia was found in 50.0% of fatal hypothermia cases and 59.0% of control cases. Hyperaemia is a typical post-mortem finding in organic tissue arising from cardiovascular dysfunction prior to death. Therefore, it cannot be regarded as an indicator of hypothermia.

Vacuolization in cells of the anterior pituitary gland in hypothermia cases has been reported [2,3,17]. Ishikawa et al. [8] even reported the significance of this histopathological finding, with 40.0% vacuolization in fatal hypothermia cases and only 1% in control group cases. In the present study, vacuolization as an indication for pre-mortal hypothermic influences could not be confirmed. Nevertheless, it must be considered that autolytic alterations to the tissue could be associated with the disappearance of cellular structures that would impede further evaluation, especially as brain tissue is very vulnerable to autolytic and putrefactive changes after death.

Of the 34 pituitary glands examined in the fatal hypothermia cases, LCA-positive staining was observed in only 5.9%. In contrast, 41.0% of the control group cases showed positive staining for LCA. Hammersborg et al. [27] described inflammatory reactions in brain tissue after cooling experiments on piglets, arising from breakdown of the blood–brain barrier and subsequent fluid extravasation from vessels into the tissue.

ACTH immunostaining was positive in all cases in both groups. Ishikawa et al. [11] reported differences in ACTH expression patterns in pituitary glands that were correlated with causes of death. They found low ACTH expression in cases of hypothermia as well as in cases of hyperthermia and intoxication. Analyses of immunohistochemical staining for the stress hormones adrenaline, noradrenaline and dopamine in the hypothalamus, pituitary gland and adrenal gland revealed characteristic immunohistochemical patterns for systemic stress responses to fatal hypothermia and hyperthermia [13].

Overall, the histological alterations reported in the literature (hyperaemia, haemorrhage and vacuolization) could not be found in the tissue of the anterior lobe of the pituitary gland in cases of fatal hypothermia in our autopsy material. The present findings are in accordance with the study by Büchner [6], who also did not find any specific alterations in the pituitary tissue.

Additional immunohistochemical staining for LCA, C5b-9 and ACTH could not be established as an appropriate diagnostic marker for hypothermia. Nevertheless, further immunohistochemical investigations of the thermoregulation axis, hypothalamus – pituitary gland – peripheral endocrine glands, could potentially reveal additional markers for determining the cause of death in cases of fatal hypothermia.

## Limitations of the study

This was a retrospective study on case material from daily forensic casework with all the well-known limitations of retrospective studies compared with prospective experimental studies, wherein all possible influencing variables can be controlled.

## Highlights

• The thermoregulation axis, hypothalamus – pituitary gland – peripheral endocrine gland, plays an important role in cases of fatal hypothermia.
• Histological alterations in the anterior lobe of the pituitary gland in cases of fatal hypothermia have been reported previously, including haemorrhage, hyperaemia and cellular vacuolization.
• In this follow-up study, the histological alterations reported to be specific for hypothermia were re-investigated in a study group of 34 fatal hypothermia cases.
• In addition, immunohistochemical staining (LCA, ACTH, C5b-9) was carried out to investigate whether inflammation, necrosis or endocrine dysfunction plays a role in cases of fatal hypothermia.
• As a control group, 61 pituitary glands in cases with natural and non-natural causes of death without known ante-mortem thermal stress or head trauma were investigated.
• Hyperaemia was found in 50.0% of the study group cases and 59.0% of the control group cases. Vacuolization of cells was verified in one case in the study group and one case in the control group. Acute or recent haemorrhage in the glandular tissue was not detected. There were no signs of inflammation in the study group cases. ACTH immunostaining was positive in all cases in both groups with similar expression patterns.
• Taken together, the previously reported specific findings in the anterior lobe of the pituitary gland in fatal hypothermia could not be confirmed.
